# Extroversion-Related Differences in Gaze Behavior during a Computer Task for Assessing Speed–Accuracy Trade-Off: Implications for Sensor-Based Applications

**DOI:** 10.3390/s23146483

**Published:** 2023-07-18

**Authors:** Laura Tosini, Ana Carolina Gomes, Daniela M. Corbetta, Fernando Henrique Magalhães, Cassio M. Meira

**Affiliations:** 1 School of Arts, Sciences, and Humanities, University of Sao Paulo, Sao Paulo 03828000, Brazil; 2 Department of Psychology, University of Tennessee, Knoxville, TN 37996, USA

**Keywords:** personality, visual search, motor performance, motor control, Fitts’ law

## Abstract

The principle of Fitts’ law explains that the difficulty of movement increases when targets are farther away and narrower in width, particularly when touching two parallel targets as quickly as possible. Understanding the differences in motor and gaze behaviors between extroverts and introverts when performing tasks that require speed and accuracy is crucial for the development of sensor-based interfaces for games and rehabilitation. This study aimed to investigate such differences in a computer task that assesses the speed–accuracy trade-off (Fitts’ task). Twenty introverts and seventeen extroverts wore an eye tracker and an accelerometer attached to their hand while performing 12 trials through six levels of difficulty presented on a computer screen. The results showed that introverts had longer visual fixations at the higher difficulty levels and reduced pupil diameter variability when difficulty was intermediate, suggesting that their gaze behavior may be different from that of extroverts. However, no significant differences were found in the speed and accuracy performance or kinematic variables between extroverts and introverts. These findings have important implications for the design of interventions that require both speed and accuracy in movement, such as in the development of virtual reality/games for rehabilitation purposes. It is important to consider individual differences in motor and gaze behaviors, particularly in those who may struggle with longer visual fixations, for the design of sensor-based applications and to promote successful interventions and recovery.

## 1. Introduction

Individual differences in personality have been identified as factors that can influence how people perceive and engage with their environment, which in turn can impact their social behavior, health, and well-being. For example, extroverts typically gravitate towards vigorous and fast-paced physical activities, while introverts may prefer moderate activities without time constraints. These behavioral predispositions, which involve consistent thinking, feeling, and acting across various situations, can also influence task performance [1,2,3]. Furthermore, cortical arousal has been identified as a biological correlate of extroversion; optimal levels of arousal are essential for fundamental cortical processes such as perception, reasoning, and memory. If arousal levels exceed or fall below extreme thresholds, the point of transmarginal inhibition, which is a protective response of the body, may be surpassed [1]. These distinct neural characteristics associated with social behavior, vitality, and energization are typical of extroverts who feel comfortable with intense stimuli, while introverts tend to be more reserved and inclined to avoid strong stimuli [1,4,5]. Introverts exhibit shorter reaction times, whereas extroverts demonstrate shorter movement times [6,7,8,9]. Additionally, introverts tend to maintain attention for shorter periods [10], display higher accuracy in dart throwing [11], and exhibit superior manual dexterity [12]. Finally, it was reported that extroverts are more inclined to prioritize speed over accuracy, while introverts tend to prioritize accuracy over speed when performing a continuous rotary pursuit task, such as chasing a beam of light with a hand stylus [13]. These differences underscore the impact of personality factors on the design and implementation of sensor-based devices involving human–computer interactions, such as virtual reality games for leisure, e-sports, or rehabilitation purposes. For example, the design of sensor-based devices in rehabilitation settings, including virtual games and assistive devices, should consider how personality differences such as those between extroverts and introverts influence individuals’ perception and interaction with specific environments and devices like computer screens, mouse, accelerometers, eye-tracker glasses, and joysticks.

The speed–accuracy trade-off paradigm, as mathematically described by Fitts [14], has been widely studied, especially in aiming tasks. The task requires an individual to alternately tap two parallel targets as quickly as possible with their dominant hand. Fitts’ mathematical formulation characterizes an index of difficulty (ID) where the average movement time is linearly related to the ratio of the distance or amplitude (A) between the two targets and their width (W). The index of difficulty, or movement time, increases when the amplitude between the targets increases and/or when the widths of the targets decrease. This speed–accuracy trade-off has been observed in a wide range of situations, environments, and among different populations, including those pertinent to human–computer interaction, ergonomics, and different types of motor performance across varying age groups [15]. These findings suggest that tasks requiring greater accuracy may necessitate more cognitive resources and processing power, such as in the manipulation of sensors in assistive devices, playing games, or engaging in human–computer interaction. The amount of information necessary to perform these tasks is expressed in bits and calculated by the equation I = log2 N, where I is the information and N is the number of available alternatives. Fitts’ equation has been found to explain up to 90% of the variance in existing databases, indicating that it is a robust predictor of human motor performance in varied contexts, including those relevant to the design of sensor-based devices for human–computer interaction, such as for games and rehabilitation purposes.

Movement execution requires the visual system to direct gaze towards specific areas of interest to collect and process pertinent information, as well as to control the movements accurately [16,17]. Individual differences in personality traits can affect the visual–motor strategies used to direct the gaze [17,18]. Eye-tracking systems offer a means to evaluate gaze direction and gauge the effectiveness of decision-making processes by measuring various parameters such as search rate (duration and number of fixations and number of fixated areas), percentage of viewing time devoted to specific areas of interest, and pupillary response (variability of pupil diameter) [19,20,21,22,23,24]. These measurements provide valuable insights into pupil/corneal displacement, eye/head position, and orientation, thereby enabling the assessment of sensor-related quality and performance.

The present study seeks to examine the motor and gaze behaviors of extroverts and introverts as they perform an adapted Fitts’ task. Gaze control variables provide indices of perceptual and cognitive strategies during movement execution; thus, it is expected that notable differences in visual–motor strategies will be observed when investigating the gaze and motor behavior of individuals with different levels of extroversion. To date, no study has assessed gaze behavior in a speed–accuracy trade-off paradigm in the context of individual differences of personality traits. The aim of the present study is to investigate the visuomotor performance of extroverted and introverted participants during a speed–accuracy trade-off paradigm performed on a touchscreen computer (as used by Meira Jr. et al. [25]. Participants were asked to touch the targets on the screen so that their actions could be mapped with their gaze. We employed an eye-tracking system to provide fresh personality-related insights into the speed–accuracy trade-off paradigm. We also scrutinized movement kinematic variables with accelerometer sensors attached to the participants’ wrists. Our hypotheses are that extroverts would perform the task faster with more errors, shorter visual fixations, and a wider range of pupil diameter variability, while introverts would perform the task slower with fewer errors, longer visual fixations, and a narrower range of pupil diameter variability.

## 2. Materials and Methods

### 2.1. Participants

An a priori sample size calculation was performed based on data from a previous investigation [25] regarding the effects of age and personality traits on speedy–accuracy performance. For a test with statistical power of 80% (1 − β = 0.80) and α = 0.05, the sample size calculated was of 26 participants. We recruited a larger number of participants to avoid type II errors. A convenience sample of 37 university students (21.45 ± 2.83 years) from the state of Sao Paulo, Brazil, volunteered to participate.

The inclusion criteria were that all participants were between 18 and 30 years old, had normal vision and used no correction devices (spectacles or contact lenses), and reported previous experience in the use of standard personal touch screens. Participants with neurological or motor disorders or those taking medications that might somehow affect motor performance or cognition were excluded.

Participants’ average score on extroversion/introversion (0–18) was 11.67 ± 4.82. The participants were deliberately grouped by scores on the extroversion/introversion scale of the Eysenck Personality Questionnaire (EPQ), a reliable and valid tool for assessing individual differences in personality traits [26]: the Introverted Group scored 7.85 ± 2.99 and had 9 men and 11 women, while the scores of the Extroverted Group (9 men and 8 women) were 16.17 ± 1.38. Participants read and signed an informed consent form. The study was conducted in accordance with the Declaration of Helsinki for experiments involving humans and was approved by the School of Arts, Sciences, and Humanities’ Ethics Committee at the University of São Paulo (CAAE 88994818.0.0000.5390).

### 2.2. Task

The adapted Fitts’ task [14] to assess speed–accuracy trade-off had 12 variations (combinations of four target widths and three distances between them), with 6 IDs. We administered task execution on a custom desktop version of Fitts’ task developed by Victor Hugo Alves Okazaki (Londrina, Brazil), software Discrete Aiming Task v.2.0 http://okazaki.webs.com/softwares, accessed on 26 February 2023. The dimensions of targets’ width (W) and the amplitude (A) between targets were identical to the original study by Fitts [14], with targets of 6 inches long and widths of 2, 1, ½, and ¼ inches. The 3 amplitudes between targets were 2, 4, and 8 inches and the combinations of W and A formed a continuum of IDs from 1 (easiest; wider targets/shorter distance; W2 and A2) to 6 (hardest; narrower targets/longer distance; W¼ and A8). IDs 2 and 5 had two combinations (W2/A4 and W1/A2 for ID 2, and W½ /A8 and W¼ /A4 for ID 5), whereas IDs 3 and 4 had several possible combinations each (W2/A8, W1/A4, and W½ /A2 for ID 3, and W1/A8, W½ /A4, and W¼ /A2 for ID 4). We categorized the level of difficulty of the IDs as follows: ID 1 and 2 as low, ID 3 and 4 as intermediate, ID 5 and 6 as high.

Participants performed the task while comfortably sitting on an adjustable chair with their eyes aligned to the targets’ locations on the computer’s touchscreen (Dell Touch Inspiron All-In-One 5348 A20, 23-inch screen, full HD with 1920 × 1080 pixels, Intel Core i5—Dell Technologies, Hortolândia, Brazil). The distance from the computer screen to the participants’ shoulders was between 50–70 cm, depending on participants’ height. Touches on the screen were performed with the index finger of the dominant hand. Each of the 12 combinations represented a “trial” and the order of combinations’ execution was randomized. On every trial, the participants performed 20 touches (10 on each side—right and left), with a 40-s interval between trials. The instructions were identical to the ones used originally by Fitts. Each touch on the screen was considered a hit or an error.

### 2.3. Instruments

We used the Mobile Eye XG [27] to measure gaze behavior.

The EPQ was used to quantify the participants’ extroversion scores. This tool includes 88 questions to assess extroversion, neuroticism, psychoticism, and a lie scale/social desirability (misinterpretations of the answers). The extroversion scale ranges from 0 to 18 points. Participants scoring between 0 and 12 points were considered introverts and those scoring between 14 and 18 points, extroverts. Those who scored 13 were not included in the analyses. Due to the principle of independence between traits [1] the values of neuroticism and psychoticism were disregarded. Participants scoring above 11 in the lie scale/social desirability (0–22) were not included in the analysis. These cut-off values have been used in other studies that investigated extroverted and introverted adults in motor tasks [8,25,28,29,30].

**Figure 1:**
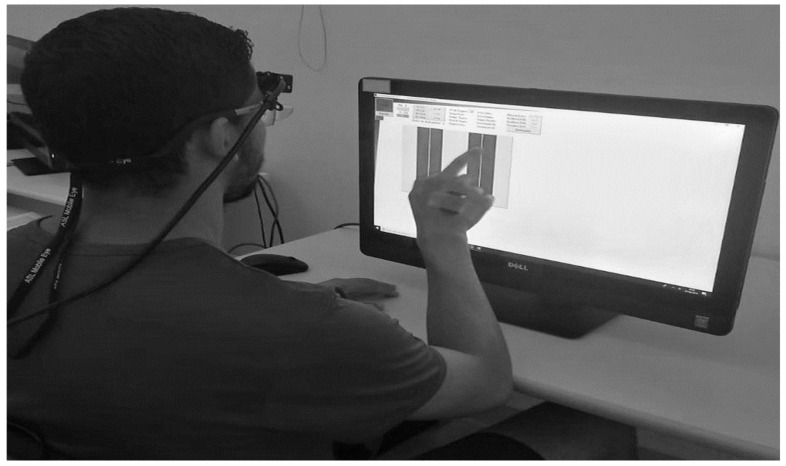
Illustration of a Participant Performing the Adapted Fitts’ Task.

For the acquisition of the kinematic variables, a Trigno Wireless Biofeedback System accelerometer (Delsys, Natick, MA, USA) was attached centrally on the back of the participant’s dominant hand (see Figure 1). The device was operated via a wireless system and data were recorded for each trial. The accelerometer was designed to easily acquire biofeedback signal detection reliably [31].

### 2.4. Procedures

Recruitment for the study was solicited in person or via electronic means. Once participants agreed to take part in the study, they filled out the informed consent and answered the EPQ. If they met the inclusion criteria (explained above), they were invited to come to the laboratory to take part in a single-session experiment.

The session initiated with the calibration of the eye tracker to ensure proper adjustment of the participants’ eye and point of regard to the scene (computer screen). While participants were sitting comfortably and looking ahead, the line adjustment and focus of the gaze were performed at nine calibration points, a prerequisite for the quality of gaze data.

Participants performed the modified version of Fitts’ task, as used in Meira Jr. et al. [25], utilizing their dominant hand to make rapid alternate touches on rectangular targets displayed on a computer touch screen. The task consisted of twelve attempts, each involving different combinations of target widths and distances between targets. These combinations were randomly generated across six levels of difficulty. The objective was to achieve maximum speed while maintaining accuracy in the touching actions.

After the researcher had read the task instructions (The instructions read to each participant prior to the familiarization trial were: “Strike these two target strips alternately. Score as many touches as you can. If you hit either of the side strips an error will be recorded. You will be given a 2 second warning before a trial. Place your hand here and start tapping as soon as you hear the buzzer. At the end of each trial I shall tell you if you have made any errors.” These instructions were identical to those used by Fitts, with the exception of the words “strip” instead of “plate” and “touch” instead of “hit”/“tap”) to the participant, an image of the task (ID 3—width 2 and amplitude 8) was shown on the screen and the participant accomplished four familiarization touches, two at each target, prior to performing the first trial. During the interval between trials, the researcher selected a new combination of width and amplitude according to a unique random order. Figure 1 depicts the experimental setting with a participant performing the task.

### 2.5. Data Analyses

We defined accuracy as the number of correct answers (a hit on the target area) in each trial. The speed (S) of each trial was calculated from the ratio between the movement time (MT) in the trial and the number of touches (20), as in the equation: S = MT/20. MT is the average time for each of the 20 movements. In addition, we ran a linear regression analysis, which equation expresses the regression line between ID and MT: MT = b0 + (b1 * ID); (b0 = intercept, b1 = slope). The coefficient of determination (r^2^) was also calculated and stands for the amount of variation in the MT explained by the regression line.

Gaze data were organized according to the following variables of interest: search rate (duration and number of fixations; number of fixated areas), percentage of viewing time (while on target, on area in between targets, or on area beyond targets), and pupil diameter variability (standard deviation of the pupil diameter mean during the trial). These variables were based on an ocular fixation (when participants casted the point-of-gaze as they looked at a stationary target in a visual field for a period equal to or greater than 100 milliseconds within 1.5 degrees of tolerance of movement—Applied Science Laboratories, 2014). Gaze data were collected at a sampling rate of 30 Hz. Video and data files were analyzed using the ASL Results analysis software.

The accelerometer collected information from the dominant hand used to touch the screen. Acceleration data were acquired at 100 Hz filtered with a 50 Hz Butterworth low-order filter. For each touch cycle (i.e., between touch n and touch n + 1), the acceleration curve was used to assess the following variables: (1) time to peak acceleration, defined as the time to achieve the maximum acceleration (i.e., the highest point of the curve for each touch cycle); (2) time to peak velocity, defined as the time between the start of each touch movement and the peak velocity [i.e., when acceleration signal crossed the zero line changing from positive (acceleration) to negative (deceleration)]; (3) time to peak deceleration, defined as the time between the start of acceleration reverse (i.e., the point where acceleration crosses zero for the first time) up to the end of the touching moment (i.e., when it returns to zero); (4) cycle duration time, defined as the time between the beginning of acceleration and the end of deceleration, until the curve has returned to zero. These variables were computed using Matlab^®^ software [version 7.12.0.635 (R2011a), MathworkInc, Natick, EUA] and an average of the 20 touch cycles for each participant was calculated and used for the statistical analyses.

The values of the three sets of measures were tabulated and organized individually, by group, and by ID pairs: low (1 and 2), intermediate (3 and 4), and high (5 and 6). The exploratory analysis revealed the existence of moderate outliers, which were kept in the analyses. Due to largely non-normal data distribution of the dependent variables (as indicated by Kolmogorov–Smirnov and Shapiro–Wilk tests) and unequal sample sizes that affect the robustness of the equal variance assumption, we ran nonparametric inferential statistics. The two groups (introverts versus extroverts) on each ID pair were compared using Mann–Whitney (Wilcoxon Rankings–U) tests. Alpha level was set at 5% for all analyses. We reported *p*-values (the probability of obtaining test results at least as extreme as the results observed) and effect sizes (the magnitude of the difference between groups–r) were calculated for significant differences.

## 3. Results

**Figure 2:**
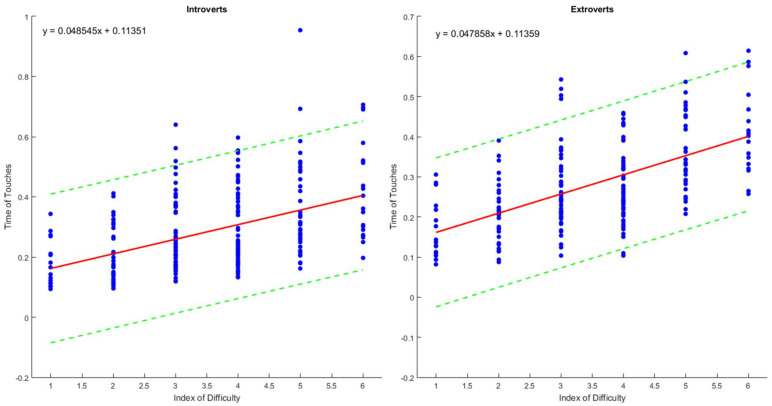
Scatterplots with 95% Confidence Intervals, Regression Lines, and Equations for Introverts and Extroverts.

The data of this study are available at https://data.mendeley.com/datasets/b5sx33fvrz/1, accessed on 26 February 2023. The results are displayed by variable sets (gaze behavior, speed–accuracy, and hand kinematics) and ID pairs (low, intermediate, and high). The descriptive data for each set of variables are shown in Table 1, Table 2, Table 3 and Table 4, whereas the regression lines are in Figure 2. All comparisons were carried out between introverts versus extroverts.

### 3.1. Gaze Behavior (Table 1)

Introverts showed longer durations of visual fixations for the intermediate IDs (U = 4421; *p* = 0.0001; r = 0.06) and high IDs (U = 1202; *p* = 0.05; r = 0.02), as well as lower pupil diameter standard deviations for the intermediate IDs (U = 5171; *p* = 0.047; r = 0.02). No significant differences were found for durations of visual fixations in the low IDs (U = 1463; *p* = 0.677) and for pupil diameter variability in the low IDs (U = 1409.5; *p* = 0.476) and high IDs (U = 1388.5; *p* = 0.402).There were no significant effects for the percentage viewing time: out of the target [low IDs (U = 1424; *p* = 0.51), intermediate IDs (U = 5892.5; *p* = 0.633), high IDs (U = 1435.5; *p* = 0.576)], left target [low IDs (U = 1489; *p* = 0.807), intermediate IDs (U = 5803.5; *p* = 0.507), high IDs (U = 1517.5; *p* = 0.941)], and right target [low IDs (U = 1501; *p* = 0.863), intermediate IDs (U = 5836; *p* = 0.551), high IDs (U = 1303.5; *p* = 0.18)].

### 3.2. Speed–Accuracy (Table 2 and Table 3, Figure 2)

The analyses did not reveal significant effects for low IDs (accuracy: U = 1516; *p* = 0.853; speed: U = 1488.5; *p* = 0.806), intermediate IDs (accuracy: U = 5925; *p* = 0.622; speed: U = 5840; *p* = 0.557), or high IDs (accuracy: U = 1452; *p* = 0.624; speed: U = 1405.5; *p* = 0.459). The regression lines (Figure 2) of the groups were represented by the equations: Introverts, y = 0.0485x + 0.1135; r^2^ = 0.9426; Extroverts, y = 0.0479x + 0.1136; r^2^ = 0.9285. No significant differences were detected for the variables of the equations (Table 3): slope (U = 158; *p* = 0.715), intercept (U = 160; *p* = 0.761), and r^2^ (U = 139; *p* = 0.345).

### 3.3. Hand Kinematics (Table 4)

The analyses showed no differences for low IDs (Time peak acceleration: U = 1271.5; *p* = 0.226; Time peak velocity: U = 1261.5; *p* = 0.204; Time peak deceleration: U = 1224.5; *p* = 0.134; Cycle duration time: U = 1242; *p* = 0.164), intermediate IDs (Time peak acceleration: U = 1242; *p* = 0.954; Time peak velocity: U = 5507.5; *p* = 0.768; Time peak deceleration; Cycle duration time: U = 5517; *p* = 0.784), or high IDs (Time peak acceleration: U = 1334; *p* = 0.512; Time peak velocity: U = 1363.5; *p* = 0.636; Time peak deceleration: U = 1332; *p* = 0.504; Cycle duration time: U = 1326; *p* = 0.48).

## 4. Discussion

This study investigated the visuomotor performance of extroverted and introverted participants during a speed–accuracy trade-off paradigm performed on a touchscreen computer. Our study provides novel evidence of extroversion-related differences in gaze behavior but not in motor performance when completing a speed–accuracy trade-off task adapted for a visuomotor task performed on a computer screen. Specifically, introverts exhibited longer visual fixations, particularly under intermediate and high levels of difficulty, indicating a prioritization of accuracy in task execution. This visual attention strategy could help prevent errors (e.g., missing a target) by gathering more detailed information from the environment. This strategy is similar to that observed in expert and team sports studies that compared visual search patterns of top-level versus non-skilled athletes, where longer fixations on the most relevant areas of the scene allowed for adjustments to anticipate action [32,33,34].

Anchoring gaze on the most relevant areas of the environment is crucial for extracting information [17,32]. In the Fitts’ task, the targets are the most informative areas, so extroverts and introverts are expected to exhibit distinct gaze patterns according to their speed and accuracy preferences, with extroverts gazing longer outside the targets and introverts focusing more on the targets. However, we did not find significant extroversion-related differences in percentage viewing time. Since the instructions emphasized accuracy over speed, extroverts adhered strictly to the instructions and could not exhibit a gaze behavior reflecting a preference for speed.

The eye-tracker sensor allowed us to measure pupillary response as a measure of cognitive effort in task performance, with variations in pupil diameter [22,35,36,37]. Results showed that the introverted group had significantly lower pupil diameter standard deviations than the extroverted group while performing the intermediate ID pair of the adapted Fitts’ task, which consisted of six combinations of the twelve possible ones (the other ID pairs had only three combinations each). This suggests a direct correlation between extroversion and the number of options available to complete a task, implying that extroverts required more cognitive effort to prioritize accuracy over speed. These findings contrast with Bækgaard et al.’s [19] results, who observed that pupil variations were associated with the onset of the task, regardless of the difficulty of the Fitts’ task performed using a head-mounted display, mouse input, head, foot, and gauze conditions. Similarly, Fletcher et al. [20] conducted research on pupil dilation during a speed–accuracy trade-off task in a sample of military personnel, with a focus on precise performance. Their results suggested that pupil size did not vary significantly as task difficulty increased, despite the increased workload. These results indicate the need for further investigation to gain a deeper understanding of the relationship between pupil response, task difficulty, and the number of options available in speed–accuracy trade-off tasks when using sensor devices, thereby enhancing our comprehension of how these factors interact and influence decision-making processes in sensor-based scenarios.

Our speed and accuracy analyses revealed no differences between extroverts and introverts, hence not supporting our hypotheses that extroverts would complete the tasks in less time but with more errors and introverts would take more time with fewer errors. Brebner’s [38] extroversion motor theory may explain these findings, as the response time between introverts and extroverts may not differ due to introverts possibly compensating for their lower speed of response organization by a more efficient analysis of the stimulus. Extroverts, on the other hand, inhibit stimulation and excitation in response organization, while introverts do the opposite. This may have allowed introverted participants to compensate for their proposed lower speed of execution by analyzing the targets efficiently at the beginning of each trial, as indicated by Bækgaard et al. study [19], thus providing insight into the role of extroversion in performing the proposed visuomotor task on the computer screen.

Our kinematic data revealed no significant differences in terms of extroversion/introversion, suggesting that the underlying mechanisms of coordination, control, feedback, and error detection/correction are similarly effective in both introverts and extroverts. Our task used online corrections and visual feedback to improve movement end-point accuracy by enabling the perception of eye-head-hand coordination to control for successful movements of reaching and touching the target centers [15]. Both groups of participants appeared to utilize similar strategies of acceleration and deceleration, potentially using the cycle time for correction strategies via intrinsic feedback. The lack of differences in terms of speed and accuracy may be attributed to the movement being performed against a vertical plane, which is different from a horizontal plane where gravity can have a greater impact on movement performance.

## 5. Limitations and Conclusions

The limitations of this study include the reliance on self-report questionnaires to assess personality, which may be influenced by various factors, and the absence of an arousal measure prior to or during task execution.

The present findings indicate that gaze behavior is a significant factor in extroversion-related differences during a speed–accuracy trade-off task, with introverts spending more time gazing (taking more time to extract visual information from the environment) and extroverts exhibiting greater variations in pupil diameter (indicating more cognitive effort).

## 6. Practical Implications

The results of the present study have important implications for the clinical and rehabilitation fields, particularly in the context of using sensor devices. Understanding the visual–motor strategies and differences between extroverted and introverted individuals during a speed–accuracy trade-off task can inform the development of targeted interventions and assistive technologies.

The fact that introverts spent more time gazing suggests that they require more time to extract visual information from the environment. This insight can be used to design rehabilitation programs that focus on improving information processing and visual attention skills in introverted individuals. Additionally, clinicians and therapists can utilize eye-tracking systems to assess and track the progress of patients with different personality traits in their motor rehabilitation.

The variations in pupil diameter observed in extroverts suggest an increased cognitive effort during the task. This finding underscores the importance of considering cognitive load and attentional demands when designing rehabilitation protocols for extroverted individuals. By understanding the specific cognitive challenges faced by extroverts, clinicians can tailor interventions to optimize their performance and reduce mental fatigue.

Therefore, the present results have important implications for the development of innovative sensor applications and sensor-based interventions that take into account the role of personality traits and gaze behavior in motor performance, ultimately improving the outcomes and quality of life for individuals undergoing rehabilitation.

## Figures and Tables

**Table 1:** Data from Introverts and Extroverts in the Low, Intermediate, and High Index of Difficulty (ID) Pairs on Visual Search Rate (Duration of Fixation–in seconds, Number (N) of Fixed Areas and Number (N) of Fixations), Percentage (P) Viewing Time (Out, Left Target and Right Target—in seconds), and Pupil Diameter (PD) Variability (in pixels).

	Introverts	Extroverts
	(Mean ± SD; 95% CI)	(Mean ± SD; 95% CI)
Low IDs		
Duration of fixation	0.50 ± 0.35; 0.42–0.6	0.39 ± 0.21; 0.33–0.45
N fixed areas	2.18 ± 0.7; 2–2.36	2.27 ± 2.04; 2.05–2.5
N fixations	15.36 ± 9.28; 12.97–17.77	16.33 ± 9.07; 13.78–18.89
P viewing time out	18.57 ± 28.69; 11.16–25.98	21.56 ± 29.75; 13.19–29.93
P viewing time left	42.74 ± 28.72; 35.33–50.17	40.30 ± 22.69; 33.92–46.69
P viewing time right	39.09 ± 28.09; 31.84–46.35	38.56 ± 29.2; 30.35–46.78
PD variability	18.59 ± 13.24; 15.17–22	20.92 ± 13.87; 17.02–24.82
Intermediate IDs		
Duration of fixation *	0.35 ± 0.25; 0.31–0.4	0.25 ± 0.08; 0.24–0.28
N fixed areas	2.65 ± 0.51; 2.56–2.74	2.71 ± 0.53; 2.61–2.82
N fixations	23.76 ± 9.49; 22.05–25.48	25.45 ± 9.27; 23.63–27.27
P viewing time out	26.28 ± 25.98; 21.59–30.98	28.20 ± 27.87; 22.73–33.68
P viewing time left	41.44 ± 17.17; 38.34–44.55	41.27 ± 18.22; 37.7–44.86
P viewing time right	32.37 ± 18.38; 29.05–35.69	30.86 ± 17.72; 27.38–34.35
PD variability *	28.66 ± 15.85; 25.8–31.53	29.39 ± 9.17; 27.59–31.19
High IDs		
Duration of fixation *	0.27 ± 0.1; 0.25–0.3	0.23 ± 0.07; 0.21–0.26
N fixed areas	2.80 ± 0.4; 2.7–2.9	2.72 ± 0.56; 2.57–2.89
N fixations	33.43 ± 10.61; 30.69–36.17	32.70 ± 11.83; 29.38–36.04
P viewing time out	33.37 ± 22.7; 27.51–39.24	39.36 ± 31.37; 30.54–48.17
P viewing time left	38.56 ± 16.34; 34.35–42.8	36.42 ± 20.87; 30.56–42.3
P viewing time right	29.23 ± 18.68; 24.41–34.06	24.82 ± 17.15; 20–29.65
PD variability	31.55 ± 19.53; 26.5–36.6	30.55 ± 13.22; 26.84–34.27

Mean ± SD: Mean ± Standard Deviations; 95% CI: 95% Confidence Interval. * Indicate significant differences between introverts and extroverts (*p* < 0.05).

**Table 2:** Data from Introverts and Extroverts on Speed (movement time/number of touches) and Accuracy (hits on the targets) in the Low, Intermediate, and High ID Pairs.

	Introverts (Mean ± SD; 95% CI)	Extroverts (Mean ± SD; 95% CI)
Low IDs		
Accuracy	19.88 ± 0.49; 19.76–20.01	19.92 ± 0.27; 19.85–20
Speed	10.12 ± 4.58; 8.94–11.31	9.86 ± 4.37; 8.63–11.1
Intermediate IDs		
Accuracy	19.17 ± 1.56; 18.89–19.46	19.22 ± 1.6; 18.91–19.54
Speed	14.61 ± 5.93; 13.55–15.69	14.19 ± 5.77; 13.06–15.33
High IDs		
Accuracy	18.18 ± 2.62; 17.5–18.86	18.76 ± 1.73; 18.28–19.25
Speed	16.29 ± 6; 14.75–17.85	15.19 ± 4.05; 14.05–16.33

Mean ± SD: Mean ± Standard Deviations; 95% CI: 95% Confidence Interval.

**Table 3:** Data from Introverts and Extroverts on Slope, Intercept, and Coefficient of determination (r^2^) obtained from the regression model applied to the values of time of touches as a function of index of difficulty.

	Introverts (Mean ± SD; 95% CI)	Extroverts (Mean ± SD; 95% CI)
Slope	0.048 ± 0.029; 0.035–0.062	0.048 ± 0.021; 0.037–0.059
Intercept	11.35 ± 9.45; 6.93–15.77	11.68 ± 8.47; 7.33–16.05
Coefficient of determination (r^2^)	73.70 ± 15.02; 66.67–80.73	74.96 ± 22.11; 63.59–86.33

Mean ± SD: Mean ± Standard Deviations; 95% CI: 95% Confidence Interval.

**Table 4:** Data from Introverts’ and Extroverts’ Kinematic variables (obtained from the accelerometer attached to the hand, expressed in seconds) for the Low, Intermediate, and High ID Pairs.

	Introverts (Mean ± SD; 95% CI)	Extroverts (Mean ± SD; 95% CI)
Low IDs		
Time peak acceleration	0.22 ± 0.03; 0.21–0.23	0.21 ± 0.03; 0.2–0.22
Time peak velocity	0.43 ± 0.06; 0.42–0.45	0.42 ± 0.06; 0.4–0.44
Time peak deceleration	0.65 ± 0.1; 0.62–0.68	0.62 ± 0.1; 0.6–0.65
Cycle duration time	0.87 ± 0.14; 0.83–0.91	0.83 ± 0.13; 0.8–0.88
Intermediate IDs		
Time peak acceleration	0.21 ± 0.05; 0.2–0.22	0.20 ± 0.04; 0.2–0.21
Time peak velocity	0.42 ± 0.09; 0.41–0.45	0.41 ± 0.07; 0.4–0.43
Time peak deceleration	0.63 ± 0.14; 0.61–0.67	0.62 ± 0.11; 0.6–0.65
Cycle duration time	0.85 ± 0.19; 0.82–0.89	0.83 ± 0.15; 0.81–0.87
High IDs		
Time peak acceleration	0.22 ± 0.04; 0.22–0.24	0.23 ± 0.04; 0.22–0.25
Time peak velocity	0.45 ± 0.08; 0.44–0.48	0.46 ± 0.08; 0.44–0.49
Time peak deceleration	0.68 ± 0.12; 0.65–0.72	0.70 ± 0.12; 0.67–0.74
Cycle duration time	0.91 ± 0.16; 0.88–0.96	0.93 ± 0.15; 0.89–0.98

Mean ± SD: Mean ± Standard Deviations; 95% CI: 95% Confidence Interval.

## Data Availability

The authors confirm that the data supporting the findings of this study are available at https://data.mendeley.com/datasets/b5sx33fvrz/1, accessed on 26 February 2023.
